# A Systematic Review of Effect of Prenatal Zinc Supplementation on Birthweight: Meta-analysis of 17 Randomized Controlled Trials

**Published:** 2011-04

**Authors:** Samson G. Gebreselassie, Fikre E. Gashe

**Affiliations:** ^1^Awassa College of Agriculture, Hawassa University, Ethiopia; ^2^School of Public Health, Addis Ababa University, Ethiopia

**Keywords:** Birthweight, Medical research, Meta-analysis, Impact studies, Randomized control trails, Systematic review, Zinc supplementation

## Abstract

The effect of prenatal zinc supplementation on birthweight is controversial as randomized controlled trials (RCTs) report conflicting conclusions. A systematic review which includes meta-analysis was done on 17 RCTs conducted worldwide since 1984 to assess the effect of prenatal zinc supplementation on birthweight. The studies were identified through web-based search. Heterogeneity among studies was assessed using Cochrane Q test statistic. Effect-size was measured based on standardized mean difference. Pooled effect-size was computed using a variant of random effect model. Thirteen of the 17 RCTs found no association, three reported positive association, and one reported negative association. Based on fixed and random effect models, the pooled effect-sizes were 0.0268 [95% confidence interval (CI) 0.0764, −0.0229) and 0.0712 (95% CI 0.1619, −0.0194) respectively. The effect-size estimate remains insignificant after stratification was made based on the dose of supplementation (optimal vs high dose), type of study (community vs institution-based), and type of source country (developed vs developing). The meta-analysis did not witness any association between birthweight and prenatal zinc supplementation.

## INTRODUCTION

Zinc is one of the essential trace elements and a member of the major micronutrients which have attained prominence in human health and nutrition (1). It is required for many biological functions, including DNA synthesis, cell division, gene expression, and stabilization of molecular structures. It is also vital for the activity of more than 300 enzymes participating in the metabolism of macronutrients, micronutrients, and nucleic acids (2, 3). Since its importance for human physiology was identified in 1963, many studies witnessed its crucial importance for immune function, linear growth and gain in weight, and neuropsychological and cognitive functions of human beings. Its involvement in such diverse and fundamental activities probably accounts for its essentiality for all forms of life (3).

Poor maternal zinc status is associated with adverse pregnancy outcomes as zinc is assumed to be essential for normal foetal growth and development (4). Animal experiments indicate that severe prenatal zinc deficiency is associated with spontaneous abortion and congenital malformations (5) whereas milder forms are attributed to low birthweight (LBW), intrauterine growth retardation (IUGR), and preterm delivery (6). Difficult and prolonged labour, haemorrhage, uterine dystocia, and placental abruption have also been documented in female rats fed zinc-deficient diets throughout pregnancy (7). The poor pregnancy outcomes in women with acrodermatitis enteropathica are also consistent with effects observed in zinc-deficient pregnant animals (8). However, studies that attempted to evaluate the effect of prenatal zinc status on maternal health and pregnancy outcomes in apparently healthy individuals yielded conflicting conclusions (9).

As to the effect of prenatal zinc supplementation on anthropometric indicators is concerned, of more than 20 RCTs conducted so far, few reported positive association between zinc supplementation and anthropometric measurements. According to a study in India, birthweight of infants born to women in the placebo group averaged only 2.6 kg (10). Infants born to zinc-supplemented mothers were 0.3-0.8 kg heavier, depending on the length of time supplemental zinc was provided for. If zinc supplementation was initiated in the first trimester, the effect on birthweight would be greater than if it was initiated in the third trimester (10). A study in the USA among disadvantaged African-American women reported that supplemented zinc increased birthweight by 126 g and increased head-circumference of infant by 0.4 cm (11). Another study in the USA (12) and a study in Chile (13) reported that zinc supplementation enhanced birthweight significantly—approximately by 150 and 69 g respectively. A study in Iran found significantly higher head-circumference of newborns in the supplemented group than in the control group (35.0 cm vs 33.7 cm) but no increments in birthweight (14). However, the remaining RCTs failed to witness any association between anthropometric indicators and prenatal zinc supplementation (15-25).

The objective of this systemic review and meta-analysis was to examine the effect of prenatal zinc supplementation on birthweight. A similar meta-analysis was published in 2009 by the Cochrane Collaboration Group (26). However, this analysis has included four more additional studies and provided the results based on both fixed and random effect models.

## MATERIALS AND METHODS

### Study design

This is a systematic review which includes a meta-analysis.

### Inclusion and exclusion criteria

To assure the quality of analysis, only RCTs were included in the analysis. Articles written in only English language were considered. The exclusion criteria were set based on multiple criteria, including low dose of supplementation (less than 15 mg/day), unavailability of vital information in the articles (mean and standard deviation of birthweight in intervention and control arms, percentage of LBW babies in both arms) and low level of compliance (<70%) for the supplement.

### Search strategy and evaluation of studies

Studies were mainly identified through web-based descendent (identifying key literature and look online the other studies which cite them) and ancestor (look into the references of a key article) search techniques. The study did not involve any manual search of articles or contacts with authors. The electronic search was performed within the Cochrane Library and MEDLINE databases. Further studies were identified using the Google Scholar search engine. Key combination search terms were “zinc supplementation and birthweight” and “zinc supplementation and birth outcomes”. Literature written in only English language were considered, and no limit was made on date of publication of the articles.

Initially, 21 (20 published and 1 PhD dissertation) articles and abstracts based on RCTs were located. The principal investigators reviewed all of them using the predefined inclusion criteria. They also checked quality of the studies in terms of reasonable level of compliance for the supplementation (>70% compliance), follow-up (<30% loss to followup), and assuring the comparability of the intervention and control groups based on key variables (maternal age, educational status, maternal anthropometric indicators, gestational age, parity, etc.). Disagreements were solved by repeated evaluations and discussions.

Three of the 21 RCTs, conducted in Germany (27), USA (28) and Denmark (29), were excluded as vital information, such as mean and standard deviation of birthweight in intervention and control arms and proportion of LBW babies in both arms, could not be extracted from the articles and abstracts. A study in South Africa was also excluded since it used very low dose (4.3-12.9 mg/day) of zinc supplementation (30). Hence, the analysis was done with the remaining 17 studies.

From each of the studies included, information on total number of zinc-supplemented and control children, number of LBW babies (<2,500 g), mean birthweight and standard deviation (SD) for both arms, nature of the population, and dose and duration of supplementation were extracted.

### Analysis of data

The description of original studies was made using frequency and forest plot. Heterogeneity among studies was statistically assessed using Cochrane Q test statistic. The test statistic indicated random heterogeneity among studies (p=0.09). Hence, random effect model was used in the analysis. Among studies, variation was assessed using DerSimonian and Laird's (DL's) estimator. To control the effect of dose of supplementation (optimal or high dose), type of the study (community or institution-based), and type of source country (developed or developing), stratified analysis was made. We analyzed data using Metaeasy add-in for the MS Excel software (version 1.0). As relatively fewer articles were included in the analysis, Funnel plot was not used for assessing publication bias.

The strength of association between zinc supplementation and birthweight was assessed using effect-size which measures the strength or magnitude of difference between two sets of data (in this case treatment groups). It is the difference between the mean values of the two groups, divided by the standard deviation. The larger the effect-size, the greater is the difference or impact of an intervention. Cohen proposed operational definitions of 0.2, 0.5, and 0.8 as small, medium, and large effect-sizes respectively (31).

## RESULTS

### Description of original studies

Four studies from the USA, three from the UK, six from Asian countries, three from Latin American countries, and only one from Africa were included in the analysis. In total, these studies involved 6,209 pregnant women in intervention and control arms. The sample-size of the studies ranged from 1,075 in Nepal (32) to 52 in the UK (18). With the exception of six community-based studies, all were health institution-based. All the studies randomized the study subjects into intervention and control groups around the mid-point of the second trimester (16-20 weeks). The dose of the supplement in 12 studies was 15-25 mg/day. However, in the remaining five studies, a higher dose (25-62 mg/day) of supplementation was used. All the studies were published in the last 25 years (1984-2009). The basic information of each specific study is presented in Table 1.

**Table 1.** Description of 17 randomized controlled trials on the association between prenatal zinc supplementation and birthweight, 1984-2009

Author and year of publication	Country	Dose	Type	Intervention Group		Control group		Mean difference (g) (95% CI)	Total sample-size
				Mean birthweight (g) (SD)	Samplesize	Mean birthweight (g) (SD)	Sample-size		
Goldenberg RL et al., 1995 (11)	USA	25 mg	HIB	3,214 (669)	294	3,088 (728)	286	126 (12.1, 239.9)	580
Hunt IF *et al*., 1985 (15)	USA	20 mg	HIB	3,352 (430)	55	3,338 (592)	51	14 (-184.3, 212.3)	106
Tamura T *et al*., 2003 (12)	USA	25 mg	HIB	3,267 (592)	173	3,117 (666)	182	150 (19.1, 280.9)	355
Hunt IF *et al*., 1984 (16)	USA	20 mg	HIB	3,430 (509)	81	3,368 (442)	64	62 (-93.0, 217.0)	145
Mahomed K *et al*., 1989 (17)	UK	20 mg	HIB	3,291 (581)	247	3,319 (531)	244	-28 (-126.4, 70.4)	491
Simmer K *et al*., 1991 (18)	UK	23 mg	HIB	2,990 (540)	30	2,820 (600)	22	170 (-146.5, 486.5)	52
Robertson JS *et al*., 1991 (19)	UK	62 mg	HIB	3,264 (722)	72	3,220 (871)	62	44 (-229.5, 317.5)	134
Caulfield LE *et al*., 1999 (20)	Peru	15 mg	CB	3,267 (461)	488	3,300 (498)	469	-33 (-93.9,27.9)	957
Merialdi M *et al*., 2004 (21)	Peru	25 mg	CB	3,351 (427)	94	3,319 (389)	101	32 (-82.9,146.9)	195
Hafez A *et al*., 2004 (22)	Pakistan	20 mg	HIB	3,023 (456)	121	3,061 (444)	121	-38 (-151.4,75.4)	242
Christian P *et al*., 2003 (32)	Nepal	25 mg	CB	2,598 (428)	553	2,652 (429)	522	-54 (-105.3,−2.7)	1,075
Danesh A *et al*., 2009 (14)	Iran	30 mg	HIB	2,961(498)	42	2,819 (609)	42	142 (-95.9,379.9)	84
Dijkhuizen MA *et al*., 2004 (23)	Indonesia	25 mg	CB	3,200 (500)	84	3,150 (400)	80	50 (-88.3, 188.3)	164
Saaka M *et al*., 2009 (24)	Ghana	40 mg	HIB	3,105 (490)	272	3,120 (486)	271	-15 (-97.1, 67.1)	543
Duran CC *et al*., 2001 (13)	Chile	20 mg	CB	3,319 (460)	249	3,250 (514)	258	69 (-15.8, 153.8)	507
Osendarp JM *et al*., 2009 (25)	Bangladesh	30 mg	CB	2,513 (390)	194	2,554 (393)	216	-41 (-116.9, 34.9)	410
Garg HK *et al*., 1993 (10)	India	45 mg	CB	3,000 (513)	106	2,600 (541)	62	400 (233.6, 566.4)	168

CB=Community-based

CI=Confidence interval

HIB=Health institution-based

SD=Standard deviation

### Pooled effect-size

The effect-size estimate of the specific studies showed that the majority (n=13) of the studies found no association between zinc supplementation and birthweight. Three studies reported positive association while one study reported negative association. Based on fixed and random effect model assumptions, the mean (pooled) effect-sizes were 0.0268 (95% CI 0.0764, −0.0229) and 0.0712 (95% CI 0.1619, −0.0194) respectively. In both the cases, the meta-analysis showed no significant association between birthweight and zinc supplementation. The specific and pooled effect-sizes are presented in the figure.

To control the possible effect of confounders, stratification was made based on type of country (developed or developing), dose of supplementation (optimal or high dose), and type of study (health institution-based or community-based). The classification into developed or developing country was made according to the World Bank cut-off point of gross national income per capita of US$ 3,945 (33). The mean effect-sizes, based on the fixed and random effect models for the respective categories, are summarized in Table 2.

**Table 2.** Mean effect-size in different categories of studies: 17 randomized controlled trials on the association between prenatal zinc supplementation and birthweight, 1984-2009

Stratifying variable	Total sample-size of studies	Effect-size with 95% CI (fixed effect model)	Effect-size with 95% CI (random effect model)
Type of country			
Developed	1,863	0.107 (0.197, −0.017)	0.107 (0.199, −0.016)
Developing	4,345	-0.008 (0.051, −0.068)	0.057 (0.183, −0.070)
Type of study			
Community-based	3,476	0.101 (0.174, 0.026)	0.129 (0.267, −0.010)
Health institution-based	2,732	-0.031 (0.041, −0.103)	0.075 (0.268, −0.118)
Dose of supplementation			
15-25 mg/day	4,869	0.016 (0.072, −0.040)	0.040 (0.123, −0.043)
26-62 mg/day	1,339	0.067 (0.175, −0.041)	0.169 (0.457, −0.119)

CI=Confidence interval

In all the categories, the Q test statistic was 0.5-0.05 and, as such, the random effect model was taken as the appropriate estimator of pooled effect-size. The stratification indicated that prenatal zinc supplementation was not associated with birthweight, irrespective of dose, type of study, and country.

## DISCUSSION

Another meta-analysis of 14 RCTs conducted by Cochrane group also failed to show any association between prenatal zinc supplementation and birthweight (26). According to this study, the pooled difference in the mean birthweight between the zinc supplemented and the control group was −10.59 g (95% CI −36.71, 15.54). Of 10 studies conducted in populations with low serum zinc or low dietary zinc intake, the pooled difference in the mean birthweight between the zinc-supplemented and the control group was −11.42 g (95% CI −38.82, to 15.98) (26). Of three studies conducted in population with normal zinc status or adequate dietary zinc intake, the pooled difference in the mean birthweight between the zinc-supplemented and the control group was-2.32 g (95% CI −88.94, to 84.30). In both the strata, zinc supplementation had no association with birthweight (26).

However, the finding of the two meta-analyses was not consistent to what is documented by many observational studies. In Japan, Higashi A *et al*. have demonstrated that maternal serum zinc level in the second trimester was not related to birthweight of infants (34); however, zinc status in the third trimester was an important determinant. A case-control study in Tanzania reported that mothers with low zinc levels were 2.6 times more at risk of having LBW babies compared to those with normal zinc levels, and newborns with low zinc levels were 2.8 times more at risk of being born with low weight (35). A study in the USA also found that, among both white and black mothers, serum zinc concentration was significantly related to birthweight after various independent determinants of birthweight were controlled (36). A similar association was also documented by studies in Turkey (37), India (38), and China (39).

The discrepancy between the conclusion of this analysis and the findings of many observational studies cannot be explained by non-compliance to zinc supplementation as all RCTs included in this analysis reported a satisfactory level of compliance. One possible reason might be the risk of potential confounding bias in observational studies. The other reason might be related to the bioavailability of zinc supplement. As absorption of dietary zinc can be inhibited by iron and phytate intake, the same might occur to zinc supplement. Hence, zinc level adequate to promote birthweight may not be achieved after the supplementation of zinc. This hypothesis is also supported by the findings of Hunt *et al.* in the USA (40). According to them, zinc supplementation among pregnant Hispanic women did not alter the mean zinc levels in serum or hair and did not increase the serum zinc level significantly compared to the pre-supplementation level (40).

**Fig.**
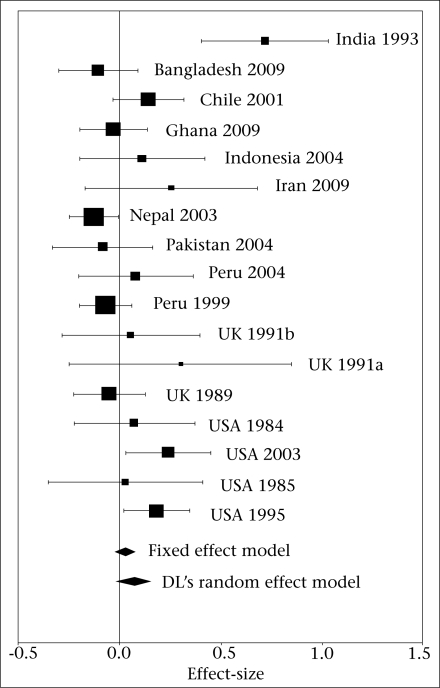
Forest plot of 17 randomized controlled trials on the association between prenatal zinc supplementation and birthweight, 1984-2009

As meta-analysis heavily depends upon published studies which are more likely to report significant results, non-significant studies which end up in the desk-drawer instead of the public domain would be systematically avoided. However, in this analysis, the problem of publication bias would be less significant as the focus of the analysis is a contemporary issue of scientific debate by which reporting any direction of association would be reasonably interesting to researchers and publishers.

### Conclusions

The study did not witness any association between birthweight and zinc supplementation. However, the finding is not conclusive as possible confounding factors were not controlled. This does not mean that zinc status is not a possible predictor of birthweight as zinc supplementation status may not perfectly correlate with serum zinc level. The bioavailability of supplemental zinc should also be investigated.

### Conflict of interest

The authors do not have any conflict of interest with regard to the findings of the study.

## References

[1] Hambidge M (2000). Zinc and health: current status and future direction. J Nutr.

[2] Prasad AS (1991). Discovery of human zinc deficiency and studies in an experimental human model. Am J Clin Nutr.

[3] Food and Agriculture Organization (2002). Zinc.. Human vitamin and mineral requirements.

[4] Shah D, Sachdev HP (2001). Effect of gestational zinc deficiency on pregnancy outcomes: summary of observation studies and zinc supplementation trials. Br J Nutr.

[5] Mambidge KM, Neldner KH, Walravens PA (1975). Zinc, acrodermatitis enteropathica and congenital malformation (letter). Lancet.

[6] Jameson S (1993). Zinc status in pregnancy: the effect of zinc therapy on perinatal mortality, prematurity and placental ablation. Ann N Y Acad Sci.

[7] Apgar J (1968). Effect of zinc deficiency on parturition in the rat. Am J Physiol.

[8] King JC (2000). Determinants of maternal zinc status during pregnancy. Am J Clin Nutr.

[9] Gibson RS (2006). Zinc: the missing link in combating micronutrient malnutrition in developing countries. Proc Nutr Soc.

[10] Garg HK, Singhal KC, Arshad Z (1993). A study of the effect of oral zinc supplementation during pregnancy on pregnancy outcome. Indian J Physiol Pharmacol.

[11] Goldenberg RL, Tamura T, Neggers Y, Cooper RL, Johnston KE, DuBard MB (1995). The effect of zinc supplementation on pregnancy outcome. JAMA.

[12] Tamura T, Goldenberg RL (1996). Zinc nutriture and pregnancy outcome. Nutr Res.

[13] Castillo-Durán C, Marin VB, Alcázar LS, Iturralde H, Ruz M (2001). Controlled trial of zinc supplementation in Chilean pregnant adolescents. Nutr Res.

[14] Danesh A, Janghorbani M, Mohammadi B (2010). Effects of zinc supplementation during pregnancy on pregnancy outcome in women with history of preterm delivery: a double-blind randomized, placebo-controlled trial. J Matern Fetal Neonatal Med.

[15] Hunt IF, Murphy NJ, Cleaver AE, Faraji B, Swendseid ME, Browdy BL (1985). Zinc supplementation during pregnancy in low-income teenagers of Mexican descent: effects on selected blood constituents and on progress and outcome of pregnancy. Am J Clin Nutr.

[16] Hunt IF, Murphy NJ, Cleaver AE, Faraji B, Swendseid ME, Coulson AH (1984). Zinc supplementation during pregnancy: effects on selected blood constituents and on progress and outcome of pregnancy in low-income women of Mexican descent. Am J Clin Nutr.

[17] Mahomed K, James DK, Golding J, McCabe R (1989). Zinc supplementation during pregnancy: a double blind randomized controlled trial. BMJ.

[18] Simmer K, Lort-Phillips L, James C, Thompson RP (1991). A double-blind trial of zinc supplementation in pregnancy. Eur J Clin Nutr.

[19] Robertson JS, Heywood B, Atkinson SM (1991). Zinc supplementation during pregnancy. J Public Health Med.

[20] Caulfield LE, Zavaleta N, Figueroa A, Leon Z (1999). Maternal zinc supplementation does not affect size at birth or pregnancy duration in Peru. J Nutr.

[21] Merialdi M, Caulfield LE, Zavaleta N, Figueroa A, Costigan KA, Dominici F (2004). Randomized controlled trial of prenatal zinc supplementation and fetal bone growth. Am J Clin Nutr.

[22] Hafeez A, Mehmood G, Mazha F (2005). Oral zinc supplementation in pregnant women and its effect on birth weight: a randomized controlled trial. Arch Dis Child Fetal Neonatal Ed.

[23] Dijkhuizen MA, Wieringa FT (2001). Vitamin A, iron and zinc deficiency in Indonesia: micronutrient interactions and effects of supplementation.

[24] Saaka M, Oosthuizen J, Beatty S (2009). Effect of prenatal zinc supplementation on birthweight. J Health Popul Nutr.

[25] Osendarp SJ, van Raaij JM, Arifeen SE, Wahed M, Baqui AH, Fuchs GJ (2000). A randomized, placebo-controlled trial of the effect of zinc supplementation during pregnancy on pregnancy outcome in Bangladeshi urban poor. Am J Clin Nutr.

[26] Mahomed K, Bhutta Z, Middleton P (2009). Zinc supplementation for improving pregnancy and infant outcome: review.

[27] Ross SM, Nel E, Naeye RL (1985). Differing effects of low and high bulk maternal dietary supplements during pregnancy. Early Hum Dev.

[28] Cherry FF, Sandstead HH, Rojas P, Johnson LK, Batson HK, Wang XB (1989). Adolescent pregnancy: associations among body weight, zinc nutriture and pregnancy outcome. Am J Clin Nutr.

[29] Jonsson B, Hauge B, Larsen MF, Hald F (1996). Zinc supplementation during pregnancy: a double blind randomized controlled trial. Acta Obstetr Gynaecol Scandinavica.

[30] Kynast G, Saling E (1986). Effect of oral zinc application during pregnancy. Gynecol Obstet Invest.

[31] Grissom RJ, and Kim JJ (2005). Effect sizes for research: a broad practical approach, 2^nd^ ed..

[32] Christian P, Khatry SK, LeClerq SC, Shrestha SR, Kimbrough-Pradham E, West KP (2001). Iron and zinc interactions among pregnant Nepali women. Nutr Res.

[33] World Bank (2010). Data: how we classify countries.

[34] Higashi A, Tajiri A, Matsukura M, Matsuda I (1988). A prospective survey of serial maternal serum zinc levels and pregnancy outcome. J Pediatr Gastroenterol Nutr.

[35] Rwebembera AA, Munubhi EK, Manji KP, Mpembeni R, Philip J (2006). Relationship between infant birth weight ≤2000 g and maternal zinc levels at Muhimbili National Hospital, Dar Es Salaam, Tanzania. J Trop Pediatr.

[36] Yosadhara P, Ramaraju LA, Rashan L (1991). Trace minerals in pregnancy: copper and zinc. Nutr Res.

[37] Cavdar AO, Söylemez FB, Cengiz B, Aydemir F (2003). Zinc status during pregnancy: a longitudinal study. J Trace Elements in Experiment Med.

[38] Neggers YH, Cutter GR, Acton RT, Alvarez JO, Bonner JL, Goldenberg RL (1990). A positive association between maternal serum zinc concentration and birth weight. Am J Clin Nutr.

[39] Ghosh A, Fong LY, Wan CW, Liang ST, Woo JS, Wong V (1985). Zinc deficiency is not a cause for abortion, congenital abnormality and small-for-gestational age infant in Chinese women. Br J Obstet Gynaecol.

[40] Hunt IF, Murphy NJ, Cleaver AE, Faraji B, Swendseid ME, Coulson AH (1989). Zinc supplementation during pregnancy: zinc concentration of serum and hair from low-income women of Mexican descent. Am J Clin Nutr.

